# Supplementary material for: The antiandrogen enzalutamide downregulates TMPRSS2 and reduces cellular entry of SARS-CoV-2 in human lung cells
Source: Nat Commun. 2021 Jul 1;12:4068. doi: 10.1038/s41467-021-24342-y (PMC8249423; doi:10.1038/s41467-021-24342-y)
Supplement: Supplementary file 1 — Supplementary Information [file 41467_2021_24342_MOESM1_ESM.pdf]

Leach et al. Supplemental Figure 1

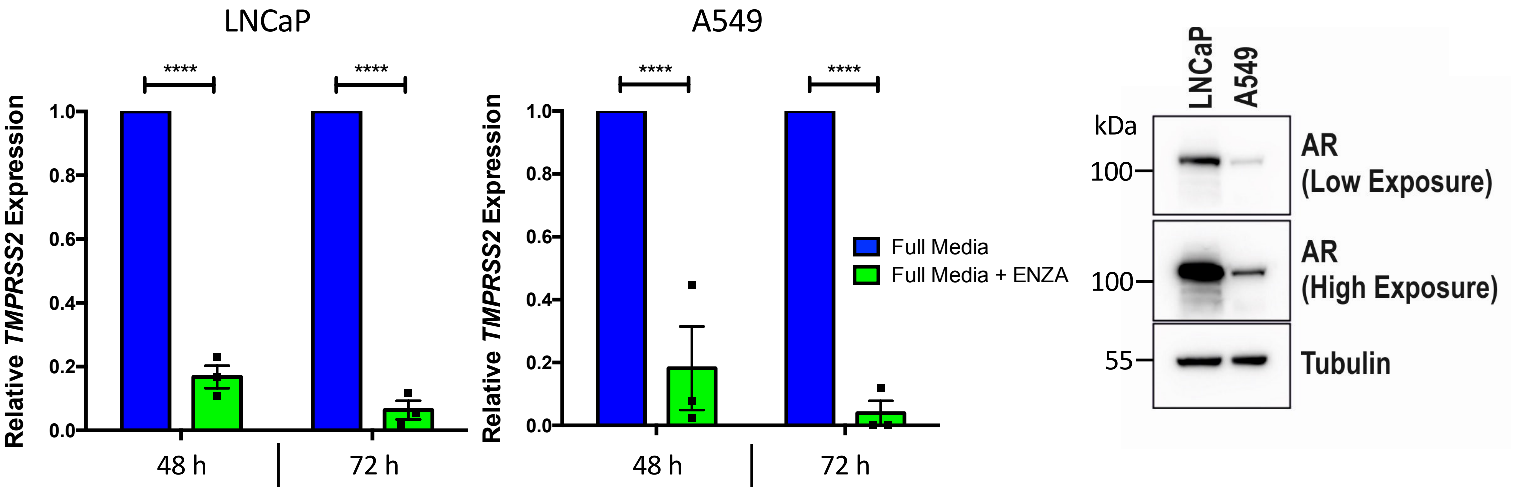

Supplemental Figure 1. ***TMPRSS2* expression is reduced by antiandrogens.** LNCaP and A549 were incubated in full media for 48 or 72 h + 10 nM mibolerone ± 10 µM enzalutamide (ENZA). RNA was harvested, reverse transcribed and qPCR performed to quantify the expression of *TMPRSS2*. Mean of 3 independent repeats (± 1SEM). Two-way ANOVA with Sidak's multiple comparison test. LNCaP 48 h $p=1.1 \times 10^{-8}$, 72 h $p=4.3 \times 10^{-9}$; A549 48 h $p=6.4 \times 10^{-5}$, 72 h $p=2.0 \times 10^{-5}$. A549 and LNCaP cells were lysed and proteins separated using SDS-PAGE. Immunoblotting was performed to visualise AR expression levels and a-tubulin was used as a loading control. Source data are provided as a Source Data file.

Leach et al. Supplemental Figure 2

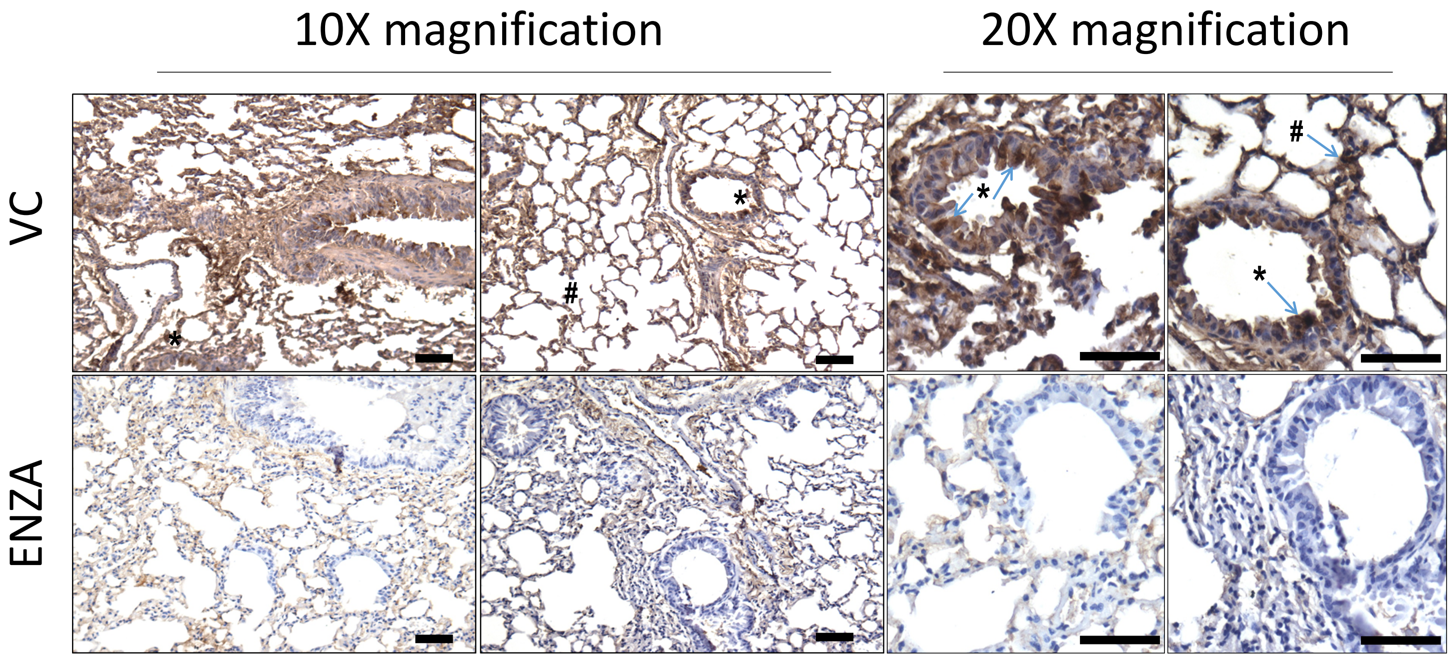

Supplemental Figure 2. Enzalutamide downregulates Ar expression in the mouse lung. Pten$^{loxp/loxp}$;Pb-Cre4 mice were treated with 50 mg/kg enzalutamide (ENZA, n=9) or vehicle control (VC, n=8) once daily for 3 days. Lungs were harvested, formalin fixed and wax embedded. Immunohistochemistry was performed using an antibody specific for the Ar. Representative immunostaining of epithelial cells (*) and parenchyma/AT cells (#). Bar = 50 µm.

Leach et al. Supplemental Figure 3

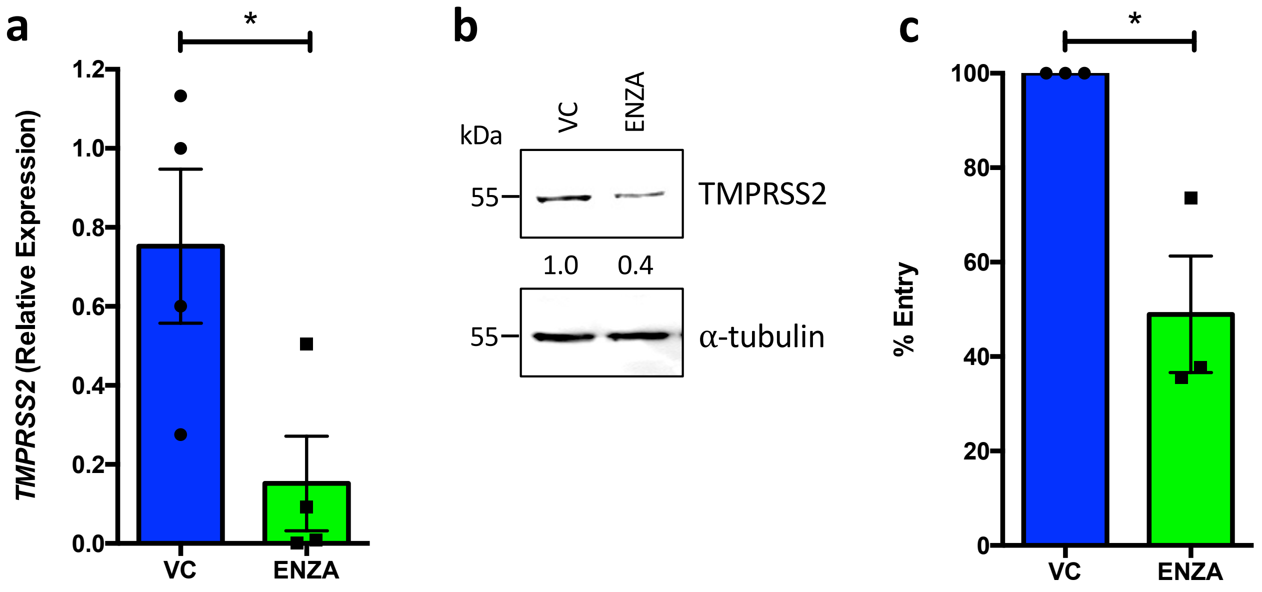

Supplemental Figure 3. Enzalutamide reduces TMPRSS2 expression and SARS-CoV-2 cell entry. A549 were grown in full media ± 10 μM enzalutamide (ENZA) for 72 hrs. **a)** RNA was harvested and qPCR performed to quantify *TMPRSS2* expression in response to vehicle control (VC) or ENZA. One-tailed t-test p=0.0197 **b)** Cells were lysed and proteins separated using SDS-PAGE. TMPRSS2 levels were visualized using immunoblotting (representative image, n=3). Densitometry was performed and TMPRSS2 expression normalised to α-tubulin levels. **c)** Cells were transduced with SARS-CoV-2 Spike protein lentiviral particles expressing luciferase. Cells were left for an additional 48 h and luciferase assays performed. Luciferase data were normalised to total protein content. Mean of 3 independent repeats ± 1SE. One-tailed t-test, p=0.0072. Source data are provided as a Source Data file.

Leach et al. Supplemental Table 1

Supplemental Table 1. Table of the chromatin areas associated with the MA0007.2 AR motif. Tabled are the 17 highest scoring motif matches in the both regulatory regions indicated in Figure 6. First column is the identifier attributed to each motif site, followed by the motif name, the chromosomal region of the motif match, statistical measures of how exact a match there is between the DNA region and the motif, and the matched sequence indicates the DNA sequence the motif matches. The p-value (two-sided) was taken from log-likelihood ratio score and assumes a zero-order null model. This value represents the probability of a random part of the motif scoring similarly to the observed match. The q-value was calculated using a Benjamini & Hochberg modification of the P-value.

Regulatory region 1 Chr21:41490000-41580500

			Start	End	Strand	p-value	q-value	Matched Sequence
**RG1a**	MA0007.2	Chr21	41498078	41498092	-	0.0000367	0.359	AAGAACTGTAAGTAC
**RG1b**	MA0007.2	Chr21	41521454	41521468	+	0.0000198	0.359	GGGCACAGCCTGAGC
**RG1c**	MA0007.2	Chr21	41521553	41521567	-	0.0000737	0.392	CAGAACAGCCTGTGG
**RG1d**	MA0007.2	Chr21	41528878	41528892	-	0.0000652	0.392	CAGAACATTATGGAC
**RG1e**	MA0007.2	Chr21	41529170	41529184	-	0.0000354	0.359	GGGCACACGCTGTTA
**RG1f**	MA0007.2	Chr21	41532582	41532596	+	0.0000215	0.359	GAGAACAGTGGGTTC
**RG1g**	MA0007.2	Chr21	41569350	41569364	-	0.000068	0.392	AGGAACACCGGGAGC
**RG1h**	MA0007.2	Chr21	41578975	41578989	+	9.64E-07	0.171	GAGAACATCGTGTGC
**RG1i**	MA0007.2	Chr21	41578977	41578991	-	3.27E-06	0.29	AGGCACACGATGTTC

Regulatory region 2 Chr21:4160000-41690500

			Start	End	Strand	p-value	q-value	Matched Sequence
**RG2a**	MA0007.2	Chr21	41605770	41605784	-	0.0000931	0.514	AGGTACAAAATGAAA
**RG2b**	MA0007.2	Chr21	41611959	41611973	-	0.0000994	0.514	AGGTACACACTGATG
**RG2c**	MA0007.2	Chr21	41630148	41630162	-	4.91E-06	0.263	AAGTACAGAGTGCGC
**RG2d**	MA0007.2	Chr21	41642129	41642143	-	0.0000122	0.269	GGGAACAGGCAGTGT
**RG2e**	MA0007.2	Chr21	41656029	41656043	-	0.0000922	0.514	AGGTACAAAGGGTAC
**RG2f**	MA0007.2	Chr21	41676130	41676144	+	0.000068	0.498	AAGAACACTGTGAGG
**RG2g**	MA0007.2	Chr21	41683746	41683760	+	0.0000209	0.367	GGGAACAGAAAGGGC
**RG2h**	MA0007.2	Chr21	41687334	41687348	+	0.0000314	0.367	CAGCACAGAGTGAAC

Leach et al. Supplemental Table 2

Supplemental Table 2. Primer sequences used in the study

**Human qPCR Primers**	**Forward (5’-3’)**	**Reverse (5’-3’)**
TMPRSS2	CCTGTGTGCCAAGACGACTG	TTATAGCCCATGTCCCTGCAG
FKBP5	ATTATCCGGAGAACCAAACG	CAAACATCCTTCCACCACAG
L19	GCGGAAGGGTACAGCCAAT	GCAGCCGGCGCAAA
GAPDH	ATGGGGAAGGTGAAGGTCG	GGGGTCATTGATGGCAACAATA

**Mouse qPCR Primers**	**Forward (5’-3’)**	**Reverse (5’-3’)**
Tmprss2	GAGAACCGTTGTGTTCGTCTC	GCTCTGGTCTGGTATCCCTTG
Ace2	TGATGAATCAGGGCTGGGATG	ATTCTGAAGTCTCCGTGTCCC
Ar	TGGGACCTTGGATGGAGAAC	CTCCGTAGTGACAGCCAGAAL19
L19	GAAATCGCCAATGCCAAC	TCTTAGACCTGCGAGCCTCA
Gapdh	GCAAAGTGGAGATTGTTGCCAT	CCTTGACTGTGCCGTTGAATTT
β-actin	CCTCTATGCCAACACAGTGC	CCTGCTTGCTGATCCACATC

**ChIP-qPCR Primers**	**Forward (5’-3’)**	**Reverse (5’-3’)**
RG1a	CCTTGCAATTGCTGACCCCA	ACAGCAAGATGGCTTTGAACT
RG1d	CATGAGGGCAGTGAGAGTGC	TTTCTCTGGTCCCAGCCATC
RG1e	CTGCTGTGTTACAAACGACG	CTCCCCGCTCTAGGATGTC
RG1f	TTACACCACTGGCTATTGGCTC	CTTTTCAGCCTTGGACATCGG
RG1g	GGCTATTGGCTCCTTCTCTTCTT	CCTGTTTCTAAACCTTTTCAGCCT
RG1h	CCAAAAGTGTTGCTCGGCTT	AGAAGTGCAGCTGGCATCG
RG2a	GCAACCTGAGCCTGTTGACT	CTCAGGTCAGGCTTCCACAC
RG2b	TCACATCTGGAGGCACTCTG	CATTGCACCTTCCGCCTTAC
RG2d	ACAGAACGGAAAGGAAGGCA	CAACTGGGAAACTGTCTGCAT
RG2e	AGTTACAACCACTGGGTGGG	AGATTCAAAGCGCCTTCACG
RG2f	ACACCCCTAGAGTATTTTCCCC	GGTGATGGTTACACTACAGCTAC
RG2g	GTAGCTCTGAGGGCAGTTCA	ATCGGATGCTGGGAATGACG
RG2h	AAGCTCCGGGGAAACCTTATT	CATGATGGCTTTTGACGCCTG
