# Supplementary material for: The antiandrogen enzalutamide downregulates TMPRSS2 and reduces cellular entry of SARS-CoV-2 in human lung cells
Source: Nat Commun. 2021 Jul 1;12:4068. doi: 10.1038/s41467-021-24342-y (PMC8249423; doi:10.1038/s41467-021-24342-y)
Supplement: Supplementary file 3 — Reporting Summary [file 41467_2021_24342_MOESM3_ESM.pdf]

nature research

Corresponding author(s): Dr Greg N Brooke and Prof Charlotte L Bevan

Last updated by author(s): 2021/05/24

Reporting Summary

Nature Research wishes to improve the reproducibility of the work that we publish. This form provides structure for consistency and transparency in reporting. For further information on Nature Research policies, see our Editorial Policies and the Editorial Policy Checklist.

Statistics

For all statistical analyses, confirm that the following items are present in the figure legend, table legend, main text, or Methods section.

n/a	Confirmed	
☐	☒	The exact sample size (*n*) for each experimental group/condition, given as a discrete number and unit of measurement
☐	☒	A statement on whether measurements were taken from distinct samples or whether the same sample was measured repeatedly
☐	☒	The statistical test(s) used AND whether they are one- or two-sided<br>*Only common tests should be described solely by name; describe more complex techniques in the Methods section.*
☒	☐	A description of all covariates tested
☐	☒	A description of any assumptions or corrections, such as tests of normality and adjustment for multiple comparisons
☐	☒	A full description of the statistical parameters including central tendency (e.g. means) or other basic estimates (e.g. regression coefficient) AND variation (e.g. standard deviation) or associated estimates of uncertainty (e.g. confidence intervals)
☒	☐	For null hypothesis testing, the test statistic (e.g. *F*, *t*, *r*) with confidence intervals, effect sizes, degrees of freedom and *P* value noted<br>*Give P values as exact values whenever suitable.*
☒	☐	For Bayesian analysis, information on the choice of priors and Markov chain Monte Carlo settings
☒	☐	For hierarchical and complex designs, identification of the appropriate level for tests and full reporting of outcomes
☒	☐	Estimates of effect sizes (e.g. Cohen's *d*, Pearson's *r*), indicating how they were calculated

Our web collection on statistics for biologists contains articles on many of the points above.

Software and code

Policy information about availability of computer code

Data collection: No software used

Data analysis:
- data were plotted and analysed using GraphPad PRISM 6
- Microarray data was processed and normalised to quartile using AltAnalyze v2.0/ Bioconductor Limma
- ChIP sequencing data (receptors in the presence of their cognate ligands) were analysed using Cistrome Analysis Pipeline (http://cistrome.org/) and visualised with WashU Epigenome Browser (v51.0.5)
- Identification of potential androgen response elements was performed using FIMO (available at http://meme-suite.org/doc/fimo.html)

For manuscripts utilizing custom algorithms or software that are central to the research but not yet described in published literature, software must be made available to editors and reviewers. We strongly encourage code deposition in a community repository (e.g. GitHub). See the Nature Research guidelines for submitting code & software for further information.

Data

Policy information about availability of data

All manuscripts must include a data availability statement. This statement should provide the following information, where applicable:
- Accession codes, unique identifiers, or web links for publicly available datasets
- A list of figures that have associated raw data
- A description of any restrictions on data availability

- For analysis of TMPRSS2 in different tissues, the GTEx dataset was used (https://www.gtexportal.org/home/).
- Transcriptomic analysis of gene expression was performed on datasets obtained from GEO: T47D cells treated with 10 nM DHT (GSE62243); data from lungs of castrated or intact mice (GSE31341).

• ChIP-Seq data was obtained from GEO: LNCaP - AR (GSE94682), H3K27ac (GSE73783), FOXA1 (GSE94682) and NR3C1/GR (GSE39880); A549 - H3K27ac (GSE29611), FOXA1 (GSE32465) and NR3C1/GR (GSE91313); MC7 - AR (GSE104399), H3K27ac (GSE94804), FOXA1 (GSE112969) and NR3C1/GR (GSE39879).
• sc-Seq - AR, TMPRSS2, and ACE2 expression were analysed using Eils Lab UCSC Cell browser (https://eils-lung.cells.ucsc.edu). Two additional single cell data sets were investigated (GSE122960)
• Figures 1A, 4A, 4B, 5A, 5C, 6C, 7A, 7B, Supplemental Figure 1, 3 have associated raw data (provided in the Source Data File).

Field-specific reporting

Please select the one below that is the best fit for your research. If you are not sure, read the appropriate sections before making your selection.

☒ Life sciences ☐ Behavioural & social sciences ☐ Ecological, evolutionary & environmental sciences

For a reference copy of the document with all sections, see nature.com/documents/nr-reporting-summary-flat.pdf

Life sciences study design

All studies must disclose on these points even when the disclosure is negative.

Sample size	• For expression analysis in cell lines and animals, at least 3 independent repeats were performed. For qPCR, all samples were also assayed in technical duplicates. As with similar studies, we find that this level of replication is sufficient to generate reproducible, statistically significant data. For ChIP-qPCR analysis, 3 independent experiments were performed. For the pseudotyped virus experiment, 3 independent experiments were performed. For the SARS-CoV-2 infection experiments, 2 independent experiments were performed in duplicate (n=4).
Data exclusions	• Individual qPCR repeats found not to amplify were set at the maximum number of cycles (40). Outliers were removed from Figure 6C (ChIP-qPCR) using Grubbs' test (alpha = 0.05).
Replication	• Independent repeats were performed and all attempts at replication were successful
Randomization	• Mice were randomly allocated to the different treatment arms
Blinding	• In mice experiments, animals were identified using unique code identifiers, and in analysis, treatments with enzalutamide or vehicle were not confirmed to the researcher until after qPCR was complete.<br>• Investigators were not blinded for the cell line experiments. However, the data collected were quantitative (qPCR, luciferase assays, TCID50) and therefore the risk of experimenter bias is negligible. Experiments were performed by different experimenters and results were found to correlate. Further, the data generated have also been verified by/correlates with the analyses performed on datasets from other groups.

Reporting for specific materials, systems and methods

We require information from authors about some types of materials, experimental systems and methods used in many studies. Here, indicate whether each material, system or method listed is relevant to your study. If you are not sure if a list item applies to your research, read the appropriate section before selecting a response.

Materials & experimental systems

n/a	Involved in the study	
☐	☒	Antibodies
☐	☒	Eukaryotic cell lines
☒	☐	Palaeontology and archaeology
☐	☒	Animals and other organisms
☒	☐	Human research participants
☒	☐	Clinical data
☒	☐	Dual use research of concern

Methods

n/a	Involved in the study	
☒	☐	ChIP-seq
☒	☐	Flow cytometry
☒	☐	MRI-based neuroimaging

Antibodies

Antibodies used	• AR (ab74272, Abcam), alpha-tubulin (B-5-1-2, Sigma Aldrich), AR (ab108341, Abcam), AR (sc-7305, Santa Cruz), b-actin (ab8226, Abcam), TMPRSS2 (ab92323, Abcam)
Validation	• anti-AR (ab74272) was used for immunoblotting and has been used in more than 67 publications, being routinely used for WB, IHC, ChIP. The antibody has been validated in multiple studies, for example using shRNA depletion of human AR (Huang et al. 2017. Cellular Physiology and Biochemistry, 43:2212–2225; Shen et al. 2017. Cellular Physiology and Biochemistry, 43:2047–2061).<br>• anti-AR (ab108341) IHC has been deemed by abcam to be suitable for IHC of paraffin embedded sections. It has been used by others for WB and IHC (e.g. Mao et al. 2019. Molecular and Cancer Therapeutics. 18, 1577-1586.<br>• anti-AR (sc-7305) has been cited in more than 450 publications and has been used for mulitple applications including WB, IF and

ChIP (e.g. Mounir et al. 2016. eLife. 5, e13964).
• The anti-tubulin antibody has been used in more than 2300 publications, routinely used as a loading control for immunoblotting. It has Sigma's "enhanced validation" status and "independent antibody verification", stated on their website (Demonstrating antibody specificity through the use of multiple antibodies against target in IHC or ICC - https://www.sigmaaldrich.com/technical-documents/articles/biology/antibody-enhanced-validation.html).
• The beta-actin antibody has been used in more than 1700 publications, routinely used as a loading control for immunoblotting. Abcam have deemed this antibody suitable for WB.
• The TMPRSS2 antibody has been deemed by Abcam to be suitable for WB and IHC of paraffin embedded sections. It has been used previously by others for e.g. WB analysis of androgen regulated genes in human prostate cells (Wang et al. 2016. Clinical Cancer Research, 22, 1531-1544) and investigation of expression in AT2 cells (Ortiz et al. 2020, EBioMedicing, 60, 102976).

Eukaryotic cell lines

Policy information about cell lines

Cell line source(s)	• LNCaP, H1944, BEAS-B, 293T, GMK and A549 were purchased from the ATCC
Authentication	• The ATCC provides STR authentication of all cell lines. Following purchase, lines were frozen at an early passage. Fresh aliquots of early passaged cells were defrosted for the experiments described here.
Mycoplasma contamination	• An early passage of cells (frozen soon after purchase) was defrosted for the experiments performed here. The ATCC confirm that cells are free of mycoplasma contamination and hence additional testing was not performed in this instance. Cells are routinely tested for mycoplasma contamination (maximum of every 3 months) using the Lonza MycoAlert Mycoplasma Detection Kit.
Commonly misidentified lines (See ICLAC register)	N/A

Animals and other organisms

Policy information about studies involving animals; ARRIVE guidelines recommended for reporting animal research

Laboratory animals	• Ptenloxp/loxp;Pb-Cre4 male mice between 20 and 30 weeks of age and between 30-37grams in weight.
Wild animals	N/A
Field-collected samples	N/A
Ethics oversight	• All work was carried out in accordance with the provisions of the Animals (Scientific Procedures) Act 1986 of the United Kingdom and under an appropriate Home Office license (PPL70_8705).

Note that full information on the approval of the study protocol must also be provided in the manuscript.
